# The Indoor Environment and Otitis Media among Australian Children: A National Cross-Sectional Study

**DOI:** 10.3390/ijerph19031551

**Published:** 2022-01-29

**Authors:** David Veivers, Gail M. Williams, Brett G. Toelle, Adriana M. Cortés de Waterman, Yuming Guo, Lyn Denison, Bo-Yi Yang, Guang-Hui Dong, Bin Jalaludin, Guy B. Marks, Luke D. Knibbs

**Affiliations:** 1 Faculty of Medicine, School of Public Health, The University of Queensland, Herston, QLD 4006, Australia; g.williams@sph.uq.edu.au; 2 Northern Clinical School, The University of Sydney, St. Leonards, Sydney, NSW 2065, Australia; 3 Sydney Local Health District, Sydney, NSW 2050, Australia; brett.toelle@sydney.edu.au; 4 Woolcock Institute of Medical Research, The University of Sydney, Sydney, NSW 2006, Australia; plantadriana@gmail.com (A.M.C.d.W.); b.jalaludin@unsw.edu.au (B.J.); g.marks@unsw.edu.au (G.B.M.); 5 Centre for Air Pollution, Energy and Health Research, Glebe, NSW 2037, Australia; yuming.guo@monash.edu (Y.G.); luke.knibbs@sydney.edu.au (L.D.K.); 6 Department of Epidemiology and Biostatistics, School of Public Health and Preventive Medicine, Monash University, Melbourne, VIC 3004, Australia; 7 ERM Services Australia, Melbourne, VIC 3000, Australia; lyn.denison@erm.com; 8 Guangzhou Key Laboratory of Environmental Pollution and Health Risk Assessment, Department of Occupational and Environmental Health, School of Public Health, Sun Yat-sen University, Guangzhou 510080, China; yangby23@mail.sysu.edu.cn; 9 Population Health, South Western Sydney Local Health District, Liverpool, NSW 2170, Australia; donggh5@mail.sysu.edu.cn; 10 Ingham Institute, Liverpool, NSW 2170, Australia; 11 South Western Sydney Clinical School, The University of New South Wales, Liverpool, NSW 2170, Australia; 12 School of Public Health, The University of Sydney, Camperdown, NSW 2006, Australia

**Keywords:** otitis media, indoor air pollution, risk factor, glue ear

## Abstract

The association between the indoor environment and lifetime prevalence of otitis media (OM) in Australian children was assessed. We analysed data from a cross-sectional study of children, aged 7–11 years, performed in twelve Australian cities during 2007–2008. The main outcome was a parental report of their child’s diagnosis with OM by a doctor. Information on the indoor environment (energy sources used for heating, cooling, and cooking, pets, and second-hand smoke exposure), in the first year of life and at present, was collected from parents by a questionnaire. Multi-level logistic regression models were used to adjust for individual- and area-level confounders. Our analysis comprised 2872 children (51% female, mean age: 10.0 (SD 1.2)). Of those, 1097 (39%) were reported to have OM. Exposure to gas heating in the first year of life was significantly associated with higher odds of OM in adjusted models (OR:1.22; 95% CI: 1.00,1.47), as was current exposure to reverse-cycle air conditioning (OR: 1.52, 95% CI: 1.27,1.82). Ownership of a cat or dog at any time was also associated with high odds of OM (OR: 1.50; 95% CI: 1.17,1.92). No other significant associations were observed. In this national study of Australian children, indoor environmental exposures associated with the lifetime prevalence of OM were gas heating, reverse-cycle air conditioning and pet ownership. Exposures in both early life and later childhood may both play a role in OM.

## 1. Introduction

Otitis media (OM) is a common childhood disorder (between 900,000 and 2.4 million cases per year in Australia) [1] in which there is acute or chronic inflammation of the middle ear space. It is responsible for substantial morbidity and health-care expenditure. It is estimated that up to 10% of primary care consultations in children in Australia are related to OM [1,2], and that the annual costs related to OM treatment in Australia are between AUD 100 and 400 million [2]. Recurrent OM is shown to cause a significant deterioration in the quality of life of both children and parents [3], as well as causing a long-term burden because of hearing loss.

The major risk factors for the development of OM in childhood have been extensively studied [4]. Commonly cited risk factors are attendance at day care [5,6,7], living in large families [5], exposure to second-hand tobacco smoke [8], the socio-economic status of the family, Indigenous status, and parental history of allergy and atopic diseases [6,9]. Genetic susceptibility has also been found [4]. Exposure to environmental factors, such as outdoor air pollution and pollen, as well as indoor sources, such as gas-fired stoves and heaters, are reported to be associated with OM in some studies; however, the findings are inconsistent [10,11,12]. These and other potentially modifiable domestic exposures, such as air conditioning for heating and cooling, as well as pets, received comparatively little attention in Australia, despite the substantial health and economic burden of OM [1,2].

In this study, we aimed to assess the associations between environmental exposures and OM in children and draw inferences regarding potentially modifiable risk factors for OM.

## 2. Materials and Methods

The Australian Child Health and Air Pollution Study (ACHAPS) was conducted during 2007–2008 in twelve Australian cities ranging in population size from 20,000 to 4.3 million [13]. ACHAPS was a national population-based cross-sectional study of Australian children aged 7–11 years and focused on the effects of long-term exposure to outdoor air pollution on respiratory health. Previous analyses of ACHAPS data showed robust associations between outdoor nitrogen dioxide (NO_2_) exposure and the presence of both asthma (current and lifetime) and impaired lung function [13,14]. 

The schools through which children were recruited into the study were selected because of their proximity to long-term air pollution monitoring stations and to provide a wide range of air pollutant exposures. Each selected monitoring station needed to have five years of reliable data and three schools in its proximity (<2.5 km). In total, 55 out of the 86 schools that were approached (64%) agreed to participate, and whole classes were then randomly selected to yield between 100 and 150 children per school. Parents were invited to participate and sent consent forms, a questionnaire and background information. No children were excluded because of race, ethnicity, or socio-demographic factors. In total, 7618 children were invited to take part. A total of 2880 (37.8%) responded. Data were collected from children living in communities of between 20,000 and 4.3 million inhabitants. Further details on the study design and questionnaire development are available elsewhere [13,14,15]. 

### 2.1. Exposure and Outcome

The questionnaire contained 70 items and was based on the International Study of Asthma and Allergies in Childhood (ISAAC), and ISAAC was used extensively and validated in previous studies of childhood asthma. The outcome measure of interest in this study was the parental answer to the question: “Has your child ever been diagnosed as having middle ear infection/otitis by a doctor or at a hospital?” 

The questionnaire was also used to collect information on domestic indoor environmental exposures. The exposures of interest were: energy sources used for heating and cooking (i.e., gas used for cooking, gas used for heating, air conditioning used for heating and the use of wood for heating or cooking). For each of these, information was obtained on current exposure and exposure in the first year of life. Questions on pet ownership at any time were also included. Information on exposure to smoking was gathered by asking about exposure to anyone “smoking in the house” at the time of the survey, and more specifically, in the case that the mother smoked, whether this was current, during the first year of life or during pregnancy. 

Data on other potential confounders were also recorded from the questionnaire. These data included information on a child’s age and birthweight, whether or not the child was born prematurely, atopic conditions (parents, siblings, and the child), day care attendance (in the first year of life), the number of siblings in the household and parental education and employment. 

### 2.2. Spatial Covariates

Each child’s exposure to NO_2_, as a surrogate for traffic-related outdoor air pollution, was estimated using fixed-site monitoring stations, which were within 2 km of the child’s school. Details of the exposure assessment methods were described previously [14]. 

Postcode-level socioeconomic status was derived from the Australian Bureau of Statistics Index of Relative Socioeconomic Advantage and Disadvantage (IRSAD), which is derived from 25 census variables of socioeconomic advantage and disadvantage [16]. The scores in this index are standardised to a normal distribution with a mean of 1000 and standard deviation of 100, with a higher score denoting a higher socioeconomic status. 

### 2.3. Statistical Analyses

All analyses were performed using SAS v.9.4 (SAS Institute Inc., Cary, NC, USA). Univariate analyses of the association between each variable and OM were undertaken. Pearson’s chi-squared test was used for categorical variables, and Wilcoxson rank sum tests or Student’s t-tests were used for continuous variables.

Some of the variables were transformed. Exposure to wood smoke, either by a wood stove or an open fire, was combined into a single variable. Maternal atopy was defined as the presence of asthma, eczema, or allergic rhinitis in the mother. Maternal education was categorised as having finished tertiary education, secondary education, or primary school only. Similar definitions were used for paternal education and atopy. The number of siblings was categorised as none, one, or more than one. 

Hierarchical logistic regression models (SURVEY LOGISTIC procedure in SAS) were used to assess associations between exposures and OM, taking into account the stratification and clustering of the data (at city- and school-level, respectively). We used a directed acyclic graph (DAG) (Supplementary Figure S1) to select a minimal sufficient adjustment set of covariates. A DAG was constructed using available and latent variables, in order to separate confounding variables from those that were not associated with both outcome and exposure or were on the causal pathway. The main model included child’s age, gender, attendance at day care (in the first year), socioeconomic status (IRSAD), as well as maternal and paternal education. 

Sensitivity analyses were also performed by adjusting for potential risk factors for OM, including birthweight, maternal atopy, paternal atopy, number of siblings, prematurity’ and outdoor air pollution (using lifetime NO_2_ exposure). The adjustments were performed by adding each variable individually (and one at a time) to the model, and then with all variables together. 

The results of logistic regression models are presented as odds ratios (OR) and their associated ninety-five percent confidence intervals (95% CIs). A *p*-value < 0.05 denoted statistical significance.

### 2.4. Approval

Human Research Ethics Committees (HRECs) of the University of Queensland (Ref: 2006000592) and University of Sydney (Ref: 10050), the Departments of Education in each of the six jurisdictions, and the Catholic Education Office of Victoria all contributed to this study. Ethics approval for the use of the ACHAPS data for this analysis was obtained from the University of Queensland HREC (Ref: 2018000469).

## 3. Results

Our analysis was based on children with valid, completed questionnaires, representing 2880 of the 7618 children who were invited to participate (37.8% response rate). Our analysis was based on 2872 (99.7%) of those children (51% female, mean (±SD) age 10 (±1.2) years) who had complete information on indoor cooking and heating (Table 1). The socio-demographic and other characteristics of the children are also presented in Table 1. The rate of children reported as having OM was 39.4%. Sixty-two percent of the mothers had completed tertiary-level education. The rates of exposure to the relevant environmental variables are also shown in Table 1.

In univariate analyses (Table 2), current heating by an open wood fire or a wood stove (OR 1.25, 95% CI: 1.00, 1.56), current heating by reverse-cycle air conditioning (OR 1.48; CI: 1.26, 1.74), heating by gas in the first year of life (OR 1.22; CI: 1.03, 1.43) and exposure to a cat or dog (OR 1.45; CI: 1.21, 1.75) were associated with OM. OM was found to be more likely in households that were smoke free.

In the adjusted model (Table 2), current heating by air conditioning (OR 1.52; CI: 1.27, 1.82), exposure to gas for cooking or heating in the first year (OR 1.22; CI: 1.00, 1.47) and exposure to a cat or dog (OR 1.50; CI: 1.17, 1.92) were all associated with an increased probability of having OM, compared with the absence of these exposures.

Exposure to maternal smoking at any time was not associated with OM on univariate analysis, and when models were constructed with the inclusion of each of these variables in turn, there were no significant changes in the effect estimates (Table 3). There was no association found between air pollution and OM.

A sensitivity analysis (testing the model by the addition of lifetime NO_2_ exposure, prematurity, birthweight, number of siblings and maternal and paternal atopic conditions) did not appreciably change the effect estimates (not shown).

## 4. Discussion

Acute otitis media (AOM) usually starts with a viral or bacterial infection, and children are more susceptible because of their anatomically immature eustachian tube [2]. These infections are more likely in winter and when children are in greater proximity to other children (day care, large families). Other factors that may play a part, by decreasing eustachian tube function, include those causing irritation and swelling of the upper respiratory tract or middle ear mucosa, such as allergy or exposure to air pollutants.

The risk factors associated with OM are extensively studied [4,5,6,7,8,9], but there is relatively little written about the effect of indoor environmental exposures, apart from second-hand smoke [10,11,12]. Therefore, in this secondary analysis of the ACHAPS data, we sought to determine if indoor exposures were associated with OM. The heating of the home by gas or reverse-cycle air conditioning as well as pet ownership was found to be associated with a significant risk of OM, whereas there was no significant effect of smoking by the mother (either indoors or in any location). In addition to this, outdoor air pollution (estimated by exposure to NO_2_) did not appear to be associated with OM in these data. 

We found that exposure to indoor gas heating in the first year of life was associated with having OM. Indoor gas combustion is common in Australia, with 31.6% of homes using gas as the main energy source for heating. Gas combustion is associated with respiratory disease because of the increased production of NO_2_ and other pro-inflammatory pollutants [14]. There is, therefore, a plausible mechanism for the use of internal gas heating to be associated with an increased risk of OM [11].

The effects of gas heating on OM were reported in a relatively limited number of overseas studies. For example, Daigler, in a case–control study of 362 infants from New York State [11], found that wood stoves were associated with OM’ but that gas used for cooking and smoking was not. Da Costa’s case–control study of 750 children less than six years old from Mozambique [10] as well as the use of wood for cooking (OR 1.85; CI: 1.57, 2.19) or charcoal for cooking (OR: 1.50; CI: 1.39, 1.62), with OM. However, Pettigrew’s cross-sectional analysis of 904 infants from non-smoking households in the United States (Virginia and Connecticut) found no association with wood burning, kerosene heating or the use of “gas appliances”, and that attendance at day care and maternal atopy were associated with OM [12]. 

While our research found that exposure to indoor gas heating in the first year of life was associated with OM, the significance of this association was only just maintained after adjustment. We also found that there was no such association with exposure to gas heating at the time of the questionnaire. It is possible that this reflects the greater vulnerability of children in their first years of life, which is well-documented in the context of environmental exposures [17] and is similarly demonstrated in Australian studies of gas heating and asthma [18]. We did not observe an association between gas stove exposure (first year or currently) and OM, despite the combustion products being similar to gas heaters. The shorter exposure duration from cooking compared with heating may explain this, as could the relatively high prevalence of unflued gas heaters in Australia at the time of the study [19]. Although the outcome is different to OM, we note that prospective Australian studies of childhood asthma reported adverse effects of gas heating that were both independent of and stronger than the effects of gas cooking [20].

Children living in a home where reverse-cycle air conditioning is used for heating had significantly higher odds of OM in our study. This exposure has rarely been explored in previous studies. Pettigrew [12] found no association between air conditioning use and OM in their multivariate analysis. Although air conditioners do not emit pollutants indoors in the same way that gas appliances do, there are other potential ways that they may affect children. One paper postulated that house dust mite accumulation found in poorly cleaned air conditioning systems may be associated with the worsening of atopic disease [21], which in turn may be involved in OM. Another possible mechanism could involve the low humidity reached when heated air is recycled in the cooler months, which can favour bacterial or viral infections [22]. 

We found that pet ownership, of either a cat or a dog, was significantly associated with OM. The few studies on this subject reported mixed results. In a birth cohort of 397 German infants, Bergroth [23] found that children with dogs at home were protective for OM (OR 0.38; CI: 0.38, 0.81). In another birth cohort of 505 infants from Boston, Celedon [24] (OR 1.9; CI: 1.1, 3.5) found that pet ownership was associated with increased OM. In a Swedish cross-sectional study of 959 adolescents, Janson [25] found an association between pet ownership in the first year of life and non-allergic asthma (OR 2.17; CI: 1.16, 1.04). It is plausible that just as sensitisation to pets is associated with asthma [26], it may also be associated with OM. 

Smoking (at any time) was not associated with OM in this study. Systematic reviews showed an increased risk of OM in children exposed to cigarette smoke [8,27]. However, individual studies found no association [6,7,28] or protective effect [29]. Smoking may be underreported in a questionnaire leading to an underestimation of its effect, but it may also be that parents of unwell children are less likely to smoke. 

This research contributes to the knowledge base regarding indoor environmental exposures and OM. OM causes a significant health burden in Australia and further research would help to better define their role before and to inform intervention studies focused on exposure reduction strategies.

The limitations of our study included the parent-reported nature of the main outcome variable. This may have introduced recall bias, although it was previously shown that the parental reporting of ear disease is accurate when the disease or intervention is serious (tympanostomy tube insertion) or more recent [30,31]. For example, a previous study of parent-reported vs. physician-diagnosed acute OM in longitudinal analyses of 157 American children aged up to 8 years reported that the number of episodes in the last 18 months and age at first diagnosis had intraclass correlation coefficients of 0.50 and 0.79, respectively [30]. However, a simple binary outcome for OM, comparable to that used in our analysis, had a sensitivity and specificity of 76% and 63%, respectively. A larger study in Finland (n = 2512) followed from birth to age 2 years reported that, overall, parents self-reported less acute OM compared with physician-diagnosed cases in medical records (47.8% vs. 61.9%, respectively) [32]. Given our focus on the lifetime prevalence of OM, these studies are not directly applicable to our context, but they do suggest a binary outcome adequately differentiates children with and without OM and does not unduly affect the overall findings we report. We note that, if present, the misclassification of a binary outcomes tends to be non-differential and lead to the attenuation of the effect estimate.

We were not able to distinguish between acute OM, OM with effusion (OME) or recurrent AOM, and there are different spectra of risks for these conditions [29]. Despite this limitation, recent studies used parental reporting to measure the prevalence of OM [7,33]. The Raine study (Western Australian Pregnancy Cohort) [6] used parental reporting backed up by otoscopic examination by a specialist nurse. It found a parent-reported rate of a child who had otitis by the age of 3 of 36.8% (compared to our rate of 39.4%).

It is also noted that the response rate to the questionnaire was only 37.8%, which is quite low and may introduce bias. Misclassification bias is also possible for exposures in the questionnaire such as smoking, where the quoted rate of smoking may be underestimated. The time frame for the occurrence of ear infections was many years (the questionnaire picked up children who had previously had OM), and thus the acute effects of changes in air pollution or seasonal variations were not captured. This may have led to an underestimation of the effect of air pollution on OM. Being a cross-sectional analysis, it is not possible to infer causality from these data.

The main strength of the study was that information on several important risk factors for ear infection was collected and covariates were selected using a DAG. The sample size was large, the study was nationally based and there was detailed information on sociodemographic and environmental covariates. However, because the participants were selected based on school attendance at schools near pollution monitoring stations, the population was inevitably from an urban (large and small cities) environment. We are therefore unable to comment on the nature of the association we found outside of urban areas.

Finally, we did not have information regarding Indigenous status. Although the burden of OM among Indigenous children is greater than non-Indigenous children in Australia, we are therefore unable to comment on this association due the limitations of our data. It is also difficult to assess the extent to which it may be a source of confounding, which we could not control for directly, and our findings should be interpreted cautiously.

## 5. Conclusions

In this national study of Australian children, indoor environmental exposures associated with a lifetime prevalence of OM were gas heating, reverse-cycle air conditioning and pet ownership. These results add new evidence that may help modify the management of children with OM.

## Figures and Tables

**Table 1:** Characteristics of the study population and the variables of interest.

Variable	n/N (%) or Mean ± SD (N)
Socio-demographic	
Age (years)	10.0 ± 1.2 (2872)
Female	1466/2872 (51)
Index of relative socioeconomic advantage and disadvantage IRSAD	1022 ± 76 (2872)
Maternal education—tertiary;	1730/2788 (62)
secondary	932/2788 (33.5)
Day care attendance in first year of life	690/2825 (24.4)
Number of siblings—more than one;	569/2822 (20.2)
one	1016/2822 (36.0)
Atopic Illnesses	
Maternal atopic diseases	1628/2872 (56.7)
Children with asthma	781/2840 (27.5)
Children with eczema	645/2794 (23.1)
Children with any positive skin prick test	989/2078 (47.6)
Indoor Exposures	
Gas for cooking—first year of life	1732/2774 (62.4)
Gas for cooking—current	1904/2840 (67.0)
Gas for heating—first year	936/2872 (32.6)
Gas for heating—current	945/2872 (32.9)
Wood stove or open fire—first year	470/2872 (16.4)
Wood stove or open fire—current	393/2872 (13.7)
Heating reverse cycle air conditioning—first year	340/2872 (11.8)
Heating reverse cycle air conditioning—current	917/2872 (31.9)
Pet ownership (cat or dog)	2140/2830 (75.6)
Maternal smoking—first year	566/2728 (20.8)
Maternal smoking—current	546/2820 (19.4)
Smoking in house—current	260/2854 (9.1)
Air Pollution	
Lifetime NO_2_ average exposure (parts per billion)	9.3 +/− 3.3 (2847)
Outcome	
Otitis media (lifetime)	1097/2783 (39.4)

**Table 2:** Associations between indoor environmental exposures and otitis media.

Variable	Crude Model			Adjusted Model †		
	OR	95% CI	*p*-Value	OR	95% CI	*p*-Value
Gas cooking (current)	0.98	0.83, 1.15	0.77	0.92	0.80, 1.06	0.23
Gas cooking (first year of life)	0.93	0.80, 1.09	0.39	0.91	0.76, 1.11	0.35
Heating by open fire or wood stove (current)	**1.25**	**1.00, 1.56**	**0.04**	1.28	0.96, 1.70	0.086
Heating by open fire or wood stove (first year of life)	1.05	0.86, 1.29	0.63	1.05	0.82, 1.35	0.69
Heating air conditioner (current)	**1.48**	**1.26, 1.74**	**<0.0001**	**1.52**	**1.27, 1.82**	**<0.0001**
Heating air conditioner (first year of life)	1.21	0.96, 1.52	0.11	1.27	0.99, 1.63	0.052
Gas heater (current)	1.06	0.91, 1.25	0.43	1.00	0.84, 1.20	0.98
Gas heater (first year of life)	**1.22**	**1.03, 1.43**	**0.015**	**1.22**	**1.00, 1.47**	**0.045**
Cat or dog in house	**1.45**	**1.21, 1.75**	**<0.0001**	**1.50**	**1.17, 1.92**	**0.002**
Smoking in household	**0.71**	**0.54, 0.94**	**0.016**	0.71	0.67, 1.24	0.53

† Adjusted for day care attendance, maternal education, paternal education and SES status (IRSAD), age and gender. The reference category for all of the tabled variables is no exposure. Bold numbers: Significant results.

**Table 3:** Associations between indoor environmental exposures and otitis media with additional adjustment for maternal smoking †.

	Maternal Smoking								
	During Pregnancy			During Child’s First Year of Life			Currently		
	OR	95% CI	*p*-Value	OR	95% CI	*p*-Value	OR	95% CI	*p*-Value
Gas cooking (current)	0.92	0.79, 1.06	0.22	0.90	0.78, 1.04	0.16	0.92	0.80, 1.07	0.28
Gas cooking (first year of life)	0.92	0.76, 1.12	0.40	0.90	0.74, 1.10	0.30	0.91	0.76, 1.10	0.33
Heating by open fire or wood stove (current)	1.27	0.95, 1.70	0.10	1.26	0.95, 1.68	0.11	1.28	0.97, 1.70	0.08
Heating by open fire or wood stove (first year of life)	1.06	0.84, 1.35	0.61	1.05	0.82, 1.34	0.70	1.07	0.84, 1.37	0.58
Heating air conditioner (current)	**1.50**	**1.25, 1.80**	**<0.0001**	**1.50**	**1.26, 1.79**	**<0.0001**	**1.52**	**1.26, 1.82**	**<0.0001**
Heating air conditioner (first year of life)	1.23	0.97, 1.58	0.09	1.25	0.98, 1.59	0.067	1.24	0.97, 1.58	0.09
Gas heater (current)	1.01	0.85, 1.21	0.89	1.02	0.86, 1.22	0.81	1.00	0.84, 1.20	0.98
Gas heater (first year of life)	**1.25**	**1.02, 1.53**	**0.03**	**1.23**	**1.01, 1.50**	**0.04**	**1.21**	**1.00, 1.46**	**0.048**
Cat or dog in house	**1.42**	**1.12, 1.81**	**0.0049**	**1.40**	**1.10, 1.79**	**0.0071**	**1.48**	**1.16, 1.89**	**0.0022**

† Adjusted for day care attendance, maternal education, paternal education and SES status (IRSAD), age and gender. The reference category for all of the tabled variables is no exposure. Bold numbers: Significant results.

## Data Availability

The paper is a secondary analysis of ACHAPS data and does not present new data.

## Supplementary Materials

The following supporting information can be downloaded at: https://www.mdpi.com/article/10.3390/ijerph19031551/s1, Figure S1: Directed Acyclic Graph Used to Help Determine Confounding Variables can be downloaded.
