# Supplementary material for: The Indoor Environment and Otitis Media among Australian Children: A National Cross-Sectional Study
Source: Int J Environ Res Public Health. 2022 Jan 29;19(3):1551. doi: 10.3390/ijerph19031551 (PMC8835613; doi:10.3390/ijerph19031551)
Supplement: Supplementary file 1 [file ijerph-19-01551-s001.zip › ijerph-1535356-supplementary.pdf]

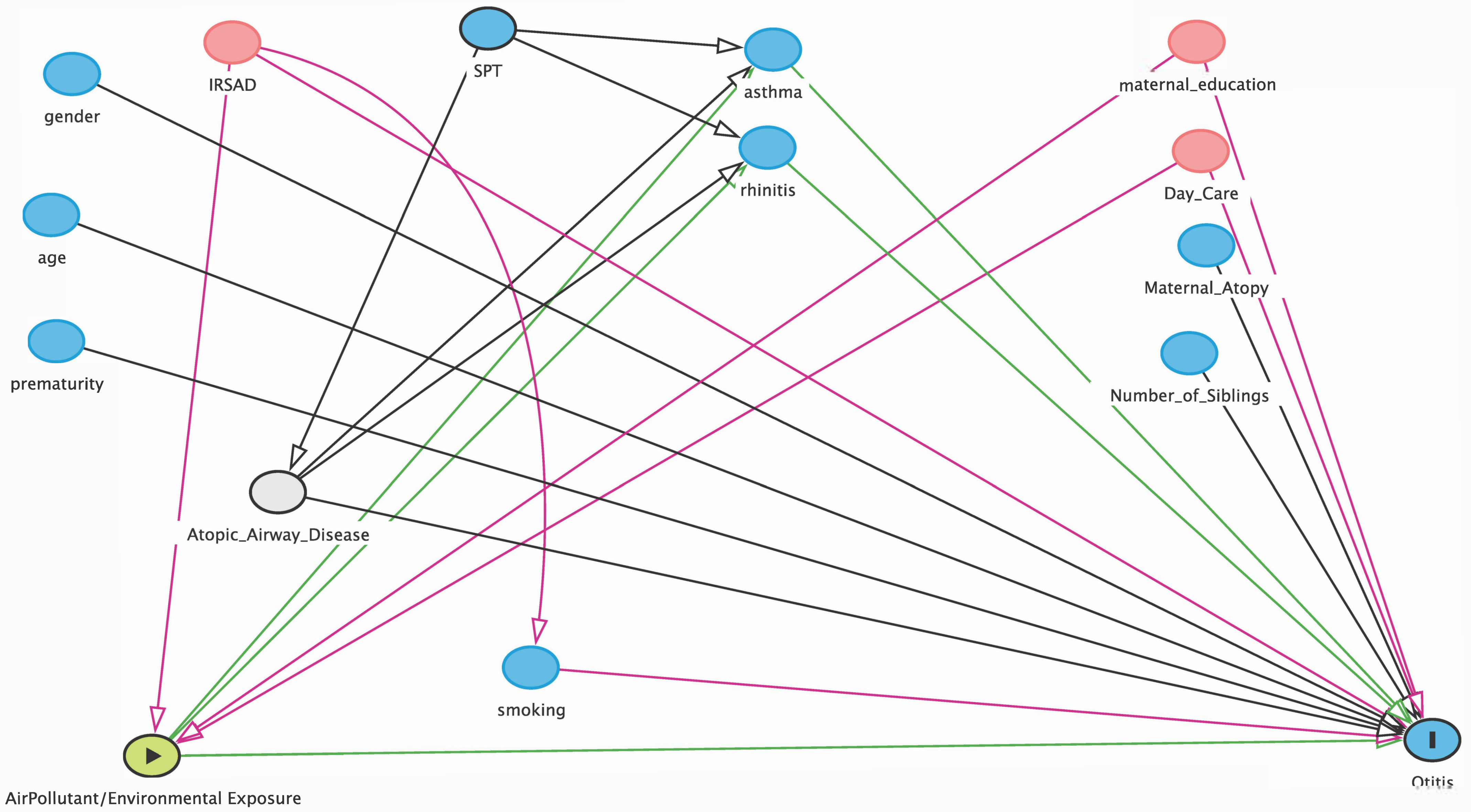

Figure S1 - Directed Acyclic Graph Used to Help Determine Confounding Variables

IRSAD - Index of Relative Socioeconomic Advantage and Disadvantage

SPT - skin prick test

" Atopic Airway Disease " an unknown variable which represents the susceptibility of an individual to suffer atopic diseases

Legend for Directed Acyclic Graph

Exposure

Outcome

Ancestor of Exposure

Ancestor of Outcome

Ancestor of Exposure and Outcome (Confounder)

Adjusted Variable

Unobserved (latent) variable

Other variable

Causal path

Biasing path
